# Institutional Responses to Drought in a High HIV Prevalence Setting in Rural South Africa

**DOI:** 10.3390/ijerph19010434

**Published:** 2021-12-31

**Authors:** Kingsley S. Orievulu, Collins C. Iwuji

**Affiliations:** 1 Africa Health Research Institute, KwaZulu-Natal, Mtubatuba 3935, South Africa; kingsley.orievulu@ahri.org; 2 Department of Global Health and Infection, Brighton and Sussex Medical School, University of Sussex, Brighton BN1 9PX, UK; 3 Centre for Africa-China Studies, University of Johannesburg, Johannesburg 2006, South Africa

**Keywords:** ART adherence, climate adaptation, drought, HIV/AIDS, South Africa, vulnerability

## Abstract

In 2015, South Africa experienced one of the worst (El Niño-induced) droughts in 35 years. This affected economic activities, individual and community livelihoods and wellbeing especially in rural communities in northern KwaZulu-Natal. Drought’s direct and indirect impacts on public health require urgent institutional responses, especially in South Africa’s stride to eliminate HIV as a public health threat by 2030 in line with the UNAIDS goals. This paper draws on qualitative data from interviews and policy documents to discuss how the devastating effect of the 2015 drought experience in the rural Hlabisa sub-district of uMkhanyakude, a high HIV prevalence area, imposes an imperative for more proactive institutional responses to drought and other climate-related events capable of derailing progress made in South Africa’s HIV/AIDS response. We found that drought had a negative impact on individual and community livelihoods and made it more difficult for people living with HIV to consistently engage with care due to economic losses from deaths of livestock, crop failure, food insecurity, time spent in search of appropriate water sources and forced relocations. It also affected government institutions and their interventions. Interviewed participants’ reflections on drought-related challenges, especially those related to institutional and coordination challenges, showed that although current policy frameworks are robust, their implementation has been stalled due to complex reporting systems, and inadequate interdepartmental collaboration and information sharing. We thus argue that to address the gaps in the institutional responses, there is a need for more inclusive systems of drought-relief implementation, in which government departments, especially at the provincial and district levels, work with national institutions to better share data/information about drought-risks in order to improve preparedness and implementation of effective mitigation measures.

## 1. Introduction

Drought recurrence in South Africa has prompted several institutional responses including government’s enactment of the Disaster Management Act 57 of 2002 (DMA) in 2003 to radically handle the effects of climate change, including drought, on the economy and people. Emergent policies like the National Drought Management Plan (DMP) (2005), National Disaster Risk Management Policy Framework (DRMPF) (2005)—including the amended Disaster Management Act in 2015—focus on robustly managing climate-related challenges. Specific to the DMA and DMP, emphases on institutional arrangements, integrated institutional capacity, disaster risk assessment and reduction planning, and response and recovery [1,2] portray the intention by government to proactively tackle drought threats through drought risk management, information management and communication, education, training, public awareness and research, as well as funding [3,4,5]. Thus, unlike many countries without adequate legislative and policy frameworks to design and coordinate drought management plans and strategies, [6] South Africa’s policy environment is enabling for dealing with drought issues [5].

Under this policy framework, different actions were required to ensure preparedness and response to drought-related processes and eventual occurrence with its associated challenges for individuals, communities, businesses and government sectors amongst others. For example, before droughts’ onset, the South African Weather Services (SAWS) analyses weather patterns and announces this onset to other departments, especially the Department of Agriculture and Rural Development (DARD) and the Department of Water and Sanitation (DWS). DARD relays this information to local extension workers who interface with local farmers [3]. The national level policies were meant to be adapted and implemented by the provincial and municipal level governments in collaboration with local partners—including private sector and civil society actors—to help rural communities, farmers and residents to better understand, prepare and manage future or present drought events [2,3]. Thus, with the arrival of the El Niño-induced drought of 2014-15/16, the presumption would be that these well-crafted policies would have effectively supported early preparedness and adequately mitigated the impact of the drought. However, with the many challenges encountered by communities and the ever-growing critique of the country’s management of the drought, many questions remain.

The recently released (2021) Intergovernmental Panel on Climate Change (IPCC) Report has reiterated the point that climate change and global warming are increasing [7]. This portends potentially catastrophic implications especially for many Low-and-Middle-Income countries lacking financial and technological resources to manage the consequences. The recent cyclone events in Southern Africa between 2019 and 2021 that led to floods in Malawi, Zimbabwe, Mozambique and even South Africa exemplify this reality. Additionally, Southern Africa is drought-prone [8,9,10], but with unchecked climate change, these recurrent drought events are projected to become even more frequent [6]. As analysts demonstrate, these climate change events, including droughts, impose varying public health challenges [3,11,12,13,14,15]. HIV-related outcomes and their effective management can therefore be directly or indirectly affected because of these recurrent drought events [16,17].

South Africa continues to carry the largest burden of the HIV pandemic despite strides made in the antiretroviral treatment (ART) programme especially the implementation of the Universal Test and Treat guidelines [18,19,20]. The context of poverty, unemployment, poor service delivery amongst other factors have continued to challenge efforts to eliminate HIV as a public health threat by 2030 following the UNAIDS target [18]. For the majority of the (poor) people in uMkhanyakude, especially people living with HIV (PLHIV), the prevalence of these sources of social vulnerability and the reality of droughts’ devastation creates competing priorities between pursuit of livelihood sustenance and time spent seeking healthcare.

Although the government and its non-governmental and multilateral partners have established and implemented various interventions to mitigate some of the effects of the drought, there are still areas of improvement. While the DMA provides a strong legal instrument for government-led responses, practical implementation shortfalls and lessons can be drawn from the experiences recorded during the 2015 drought. This is crucial in relation to issues of preparedness and inclusive planning as well as data sharing, swift implementation and response to early warning systems within and among government departments.

In this brief research note, we discuss how the devastating effects of the 2015 drought in the rural district of uMkhanyakude imposes an imperative for more concerted institutional response to climate change events with the capacity to derail progress in HIV/AIDS interventions in South Africa. Our qualitative interviews with PLHIV and government officials supporting drought-risk mitigation efforts in the district municipality showed how the 2015 droughts affected people, livelihoods and government-related activities and interventions. This provided a window to understand gaps and progress points in drought-related responses. These suggest the need for more inclusive systems of drought planning and relief implementation, and better institutional data/information-sharing about drought-risks to improve preparedness and implementation of effective mitigation measures. This paper responds to the research agenda proposed by Matthew Chersich and colleagues [12,21]. It also effectively complements biomedical HIV interventions in this high HIV prevalence setting.

## 2. Material and Methods

### 2.1. Study Setting

This study was conducted in Hlabisa sub-district, uMkhanyakude district, northern KwaZulu-Natal (KZN), South Africa. uMkhanyakude is the second largest district municipality in KZN with a predominantly rural economy centered around rainfed small-scale farming, animal husbandry, and a population that is largely dependent on government social grants and pensions [22]. It is one of South Africa’s poorest districts, with a population size >600,000, pervasive poverty and high HIV/AIDS prevalence [23].

The district is characterized by mountains, ridges, low-lying plains and fresh and sea waterways. The area is rich in biodiversity with a vast array of grasslands and nature reserves, including Hluhluwe-Umfolozi Game Reserve, Mkuzi Game Reserve, Thembi Elephant Park and Ndumu Game Reserve and St. Lucia Ocean Estuary, which serve as tourist attractions [24]. The climatic condition in uMkhanyakude is sub-tropical being mostly warm or hot during summer and mild during winter [25]. The daily average maximum temperature is projected to be >20 degrees Celsius while rainfall varies with the peak period being between January and March and ranges between 50 to 70 days per annum [25]. Climate change, including droughts, negatively impacts biodiversity, tourism and income in this district.

**Figure 1:**
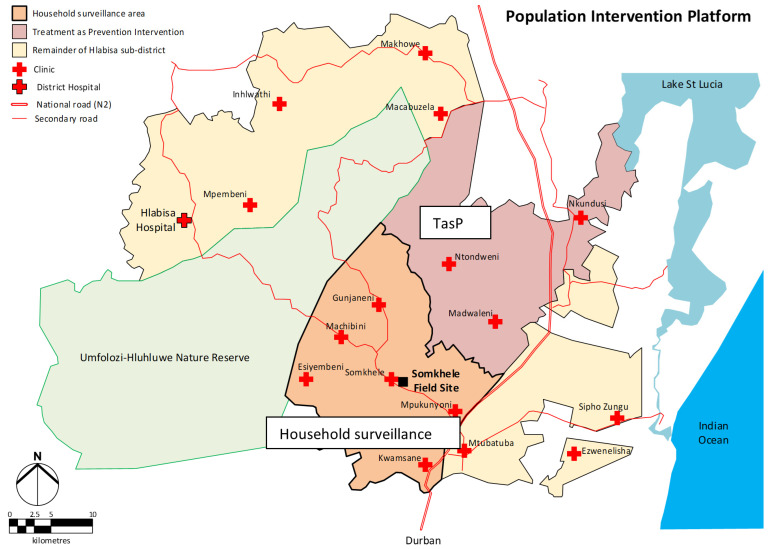
The Hlabisa sub-district showing the Africa Health Research Institute Population Intervention Platform comprising the areas denoted as TasP (Treatment as Prevention) and Household surveillance.

Between August 2019 and October 2020, we conducted fieldwork to explore the impact of drought on ART adherence among PLHIV in the sub-district within the Africa Health Research Institute’s (AHRI) population intervention platform (PIP) area (Figure 1). Study participants consisted of PLHIV (*n* = 27), government officials—Department of Health (DoH) district officials (*n* = 2) and DARD district officers (*n* = 3)—and DoH nurses (*n* = 5), hospital laboratory officers (*n* = 2) and pharmacists (*n* = 2). Sampling was random and purposive depending on the category of study participants: PLHIV were randomly recruited through the AHRI PIP while other participants were purposely recruited based on their insights about policies and interventions on topics of interest ranging from ART interventions to drought policy implementation in the district. For the purposes of this paper, we focused on the Key Informant Interviews (KIIs) conducted among DARD officials as it relates to drought policies and interventions.

### 2.2. Data Collection and Analysis

We adopted mainly qualitative research tools and techniques such as KIIs, in-depth interviews (IDIs) and telephonic interviews (TIDIs) (during the COVID-19 lockdowns from March–June 2020). TIDIs were utilized to limit physical contact in line with South African COVID-19 regulations. We conducted KIIs with government officials in English while the IDIs conducted with PLHIV were mainly in the local IsiZulu language. We used a topic guide during these interviews. The interviews spanned about 45–60 min; were audio-recorded, transcribed verbatim (and translated—for those in IsiZulu); and quality controlled by trained research assistants, a data quality coordinator and post-doctoral fellow in the Social Sciences Core department. We used Nvivo 12 Pro (QSR International, Melbourne, Australia) for data management, curation, classification, coding, and analysis. Emergent themes were organized hierarchically to capture perceptions and evaluations linked to drought impacts and policy interventions and mitigation strategies aimed to support the local population in the district. These themes were synthesized into keywords that were used to develop a systems-diagram depicting complex but plausible interactions between drought-water insecurity and ART adherence.

The data for this paper are drawn mainly from experiences of PLHIV and government officials supporting them in drought-risk mitigation within the district municipality. Other sources of data for the study and analysis were drawn from policy and legislative instruments developed within the context of South Africa’s drought-risk adaptation and mitigation framework. This study was approved by the Biomedical Research Ethics Committee (BREC) University of KwaZulu-Natal (BREC Ref: BE004/19) and the Brighton and Sussex Medical School (BSMS) Ethics Committee (Ref: ER/BSMS9B5G/2).

## 3. Results

### 3.1. Experiences and Institutional Responses: The 2015 Drought in uMkhanyakude

Our interviews with PLHIV and government officials in the uMkhanyakude district municipality revealed the extent to which the 2015 drought negatively impacted individuals, communities and government activities and efforts in varying ways.

### 3.2. Drought’s Effects on Local Societies and PLHIV

Predominantly for individuals and communities, the impacts were felt in terms of economic and livelihood losses. This ranged from the death of livestock, crop and harvest failures and devastation of families’ sources of income. These were due to depleted water sources exacerbated by both depleted soil water and lack of rainfall which culminated in the inability of crops—including weeds—to grow for production or feeding livestock. Many cows died due to thirst as interview participants recounted the severe impact of the drought on water availability and access for individual or family use. “The thing I remember the most about the drought is that cows were dying, especially when walking on the road you see that a cow has collapsed, it has lost energy. When they start saying there is a dead cow there um, you also see the cow’s body—it lost weight.” (Female, 28 years).

Experiences linked to dried rivers, dams and broken (non-operating) community taps exacerbated the challenges that people faced in uMkhanyakude during the drought. This forced some families and individuals to move their families, but more specifically their livestock, out to other areas in search of water sources (or as an attempt to escape the drought and its consequences). Even worse is the fact that individuals and cattle were forced to compete for water in the few operational dams or rivers thus creating many unhygienic and unhealthy situations for people, including PLHIV: “Sometimes it happens that there was no water for 4 months…. We will fetch water down there [where] the cows drink and defaecate in the water. We end up drinking dirty water.” (Female, 24 years).

### 3.3. Constraints on PLHIV’s Access to Treatment Care

The impact of the lack of water, as a result of both the drought and the insufficiency of government’s mitigation strategy, was felt by PLHIV in many ways. For instance, many PLHIV, especially women with care responsibilities for children, were often forced to wake up between 4am and 5am in the morning to search for distant water sources—dams, rivers or boreholes—to enable them to manage their home chores before embarking on their clinic appointments. As some of these clinics are far and many PLHIV had been forced to repurpose their income to purchase water and food within the course of the month, they either had to walk to clinics (through risky pathways, sometimes early in the morning) or forgo appointments. A participant elucidates: “I missed clinic appointment due to shortage of transport fare…. Let me put it like that. It happens that I am saving money to visit the clinic perhaps on the 20th of the month… [only] to find that money is not enough. Kids on the other side are complaining about bread. I then perhaps take this money and buy bread for them and say to myself the date … is still far. When the 20th comes, I have not been able to replace the money I took. As a result, I do not go to the clinic.” (Female, 56 years). This perspective goes to show that, the added financial pressure imposed by drought on households through increased food prices, water purchase and losses of jobs—for individuals whose employment depended on water availability—implied that many PLHIV were forced to make difficult choices whether to prioritize their economic sustenance or their HIV treatment and care. This dilemma we argue is not sustainable for progress against the elimination of HIV/AIDS as a public health threat.

### 3.4. Impacts on Government Institutions and Intervention Programmes

The interviews also highlighted that the effects of the 2015 drought were also felt by government institutions, including those mandated with the task of drought risk management. Government officials interviewed for this study highlighted that government departments, especially those working directly with local farmers affected by the drought, faced similar water scarcity-led interruptions to their work. DARD which is tasked by the DMA to house the disaster management unit that implements the DMP at district and local levels, for example, struggled to adequately provide services such as crop processing or value-addition support to farmers because there was no water available to do these. Local staff who participated in the interviews noted that they could not even manage to work effectively as there were no working ablution facilities at their offices due to water scarcity. A participant explained: “most of the time, we are… using vegetables or any kind of the harvest to do our [crop value-addition] work; to process it. When there is …[drought], people do not have … products. Then, even if they do not have [the] product … they do not have money to buy … products for training... Then, maybe so that we can proceed with our work, we have to sacrifice and buy some ingredients so that we can say we have done [the] demonstration; we have done the training.” (GOVPART-IDI-040).

Such circumstances, as the interview data show, proved difficult for individual DARD officials as regards carrying out their tasks; it also impacted the capacity of the department to provide the regular support that local farmers need. Consequently, officials had to redefine their roles and the modalities of their work at the district. These officials recounted how they paused crops value-addition (processing) support in favor of providing capacity support to people on food safety, nutrition security and water saving techniques in the context of the drought. They did these to remain relevant in their support to the local population.

### 3.5. Government Interventions: Strategies and Drawbacks

Despite these challenges, these participants stated that government, through the DARD, DWS and the local municipality, spearheaded mitigation plans and interventions. These included capacity building on water saving and cropping techniques, providing water tankers, installing borehole facilities in parts of the communities within uMkhanyakude, and providing drought-relief and compensation to farmers.

While government interventions were commendable especially towards cushioning the effects of the drought on people within this district municipality, local study participants lamented their inadequacy. This suggests that even such interventions like public awareness campaigns about the drought, compensation schemes for farmers who lost livestock or providing additional dams for livestock were not adequately visible and impactful towards ameliorating the plight of people, hence their overarching complaint about government interventions during the drought.

Similarly, our qualitative data also suggest that most of these actions were more reactive than proactive despite the intentions of the DMA and the risk management framework. There is a sense that government departments working both at the provincial and district levels were caught unawares by the drought in the area, and their communication and information sharing platforms were not effective. Participants’ statements here are insightful: “I don’t even think the department … had foreseen the drought that it will be coming on that particular [time].” (GOVPART-IDI-039)*;* “… we were alarmed by the people who were coming in the office. Farmers were reporting their loss in the fields, and then after that the department was informed…” (GOVPART-IDI-039)*;* “They walk-in—the clients used to come and report about the drought. Besides that, they also report[ed] to the tribal chiefs or Indunas or the councilors about what had happened that shows if there’s drought…” (GOVPART-IDI-040). The fact that data collection by the local DARD officials on the drought early warning indicators seemed to occur only after local farmers embarked on reporting incidents of incessant losses of livestock, crops and harvest demonstrates a late arrival to the party. The meteorological data from SAWS is a reliable source of drought information and projections, hence DARD officials may not have needed to depend on farmer-reported incidents before identifying potential dangers or potentially grasping the drought onset in the area. For example, an official speaking about gathering data on operational dams, stated:

“The information we took… was so useful such that we as the department now from an organizational perspective, we managed to see that there is a lack of dams for animals to drink because people are using water together with animals—they are competing with animals for drinking water. As you know, not everyone is able to go and buy drinking water like you and myself. So, that information assisted the department as well because after 2015 few dams were built, and few others were rehabilitated as well.” (GOVPART-IDI-041).

This excerpt buttresses the point that the data gathered by officials as early warning indicators would rather have served as reporting on the status of the impacts experienced at the local level to the province for further action. This suggests that despite the policy environment that proposes effective early warning systems that incorporate surveillance of local and grassroots vulnerabilities, [2,3] this was either non-existent or there was a failure within government to respond expeditiously. Similarly, despite officials’ avowed collaborations among government departments and between government institutions and local stakeholders through platforms such as the “Operational Sukuma Sakhe” (OSS) and engagements with advisors to the local chiefs *(Indunas)* to facilitate data/information sharing and drought planning, the experiences reported by local participants and government officials also seem to suggest an inadequate system of early warning data/information-sharing. OSS is an interdepartmental and multi-stakeholder platform for engagements, deliberations and planning pertaining to urgent issues, including health and development, in the district. It is a meeting or forum “where all the departments meet [including] NGOs, the private sector, … the community-based organizations. They … usually meet at the municipality office. That’s where we discuss everything that concerns the community including … people living with HIV and AIDS.” (GOVPART-IDI-039). This makes it a potentially effective platform for collaboration and information-sharing. However, the fact that even government departments within the district—and part of the OSS—seemed to have been caught unawares by the drought—despite its gradual processes of impact—reiterates a flawed implementation of the DRMPF. This is where both the technological advantage that SAWS provides through meteorological data projections and local surveillance (through other stakeholder-engagement) would have helped with better anticipation of the drought and its possible impacts. Although the DRMPF proposed a robust information-sharing and surveillance system, in practice what was set in motion involved a complex system of reporting between different government portfolios and stakeholders, including communities, which stalled rather than facilitated effective implementation [3,5]. The consequences were therefore dire for individuals, including PLHIV, and societies in rural KZN during the 2015 drought.

What these demonstrate is that from both institutional and community level experiences of the 2015 drought, there is a need for an intensive re-evaluation to enhance future preparedness. This is largely in terms of an institutional review, planning and execution of interventions that take into cognizance important questions of early warning systems, data sharing within and between different government institutions and complementarity between these institutions working to enhance effective drought management systems.

## 4. Discussion

### Implications for PLHIV in uMkhanyakude

HIV treatment and care requires consistency and discipline to maintain medication effectiveness for PLHIV to prevent virological failure and drug resistance which increases morbidity and mortality for individuals and is costlier for the broader economy [26]. As the HIV treatment literature demonstrates, barriers to effective ART adherence are very interconnected [27,28,29]. For instance, the economic situation of PLHIV can impact their food security, access to health facilities, access to sufficient water, or their quest to seek out employment—all of which can impose an adverse effect on optimal adherence [19,30,31,32]. Their contextual vulnerabilities—poverty, unemployment, lack of basic services can exacerbate the intensity of the challenges they face, including effectively managing their treatment [27,28,30].

The pre-eminence of drought within uMkhanyakude, as our study demonstrated, contributed negatively by intensifying precarious living conditions for people in this high HIV prevalence setting. Like many other poor people in uMkhanyakude, PLHIV faced serious socioeconomic challenges because the drought destroyed their sources of livelihood and food security. Due to these losses of livestock, crops, and water scarcity, their access to income was limited. This negatively impacted their capacity to purchase food and water for themselves and their children. The collateral effect of drought is evident: the persistent hunger and insufficient access to water or the money to procure these items would affect their likelihood to continue to engage with care. Similarly, PLHIV who were forced to relocate in search of water sources or employment were faced with the risk of missing clinic appointments or dropping out of care.

**Figure 2:**
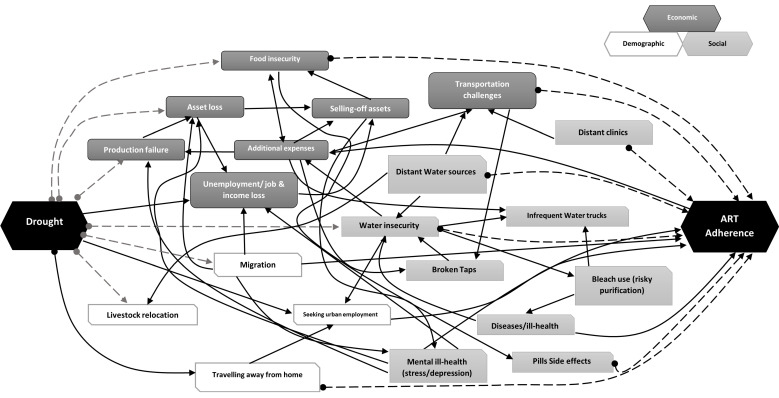
Systems diagram showing the interconnectivities between drought and treatment adherence as conceptualized from interviews conducted with study participants. The grey- and black-dotted arrows show factors directly caused or influenced by the drought, and those that HIV treatment adherence was sensitive to. The continuous black lines designate the interconnections between and among all the factors associated with drought and HIV ART adherence.

As Figure 2 shows, drought-imposed water insecurity is linked to a variety of situations tied to suboptimal HIV treatment and care. It is at the center of out-migrations from communities; PLHIV, community members and livestock sharing or competing over scarce water sources like operational dams while also using dangerous water purification strategies (like bleach) to make sure they can use it; PLHIV being forced to walk long distances in search of water sources—boreholes, rivers, dams—for home use; women living with HIV and girls faced with the inability to manage their sanitary and hygiene needs; PLHIV arriving late to clinics because they were exhausted from waking up so early to search for water and also having to walk long distances to clinics; people’s crops and livestock dying due to lack of water, thus impacting their economic well-being; government intervention systems not sufficiently implemented for water-relief and management for people and communities; and in fact, PLHIV failing to take their medication because they lacked portable drinking water and could not afford to buy bottled water from the supermarkets. In all of these, the fact that water insecurity, and related effects, can draw primarily from drought reinforces how the 2015 drought contributed to increasing the risk of suboptimal adherence.

Consequently, institutional responses to drought and HIV treatment and care must pay attention to the integrated nature of these variables as it concerns PLHIV in poor settings. “Systems thinking” has been applied in different studies to demonstrate the many connections there are between climate change related exposure variables like droughts and different health and wellness outcomes—from mental health to overall wellbeing [13,14,15,33]. Central to systems thinking is the idea that there are many indirect (socio-economic, political and psychological) linkages existing between the main causal variable, for example drought as in this South African case, and an eventual outcome such as suboptimal ART adherence. From the purview of the reported impacts of the 2015 drought on government institutions, rural societies and individuals, especially PLHIV, it is evident that many intermediate factors converge in this context to heighten the risks facing PLHIV in this poor rural setting. This also means that institutional responses cannot treat drought, HIV/AIDS and their consequent impacts in isolation, but appreciate the interconnectivities at play here. Such systems thinking, [13,14,15] especially of the drought-HIV treatment adherence nexus, provides a crucial insight in conceptualizing disaster risk management and preparedness approaches since both drought and high HIV prevalence are existential realities in the uMkhanyakude district municipality.

## 5. Conclusions and Recommendations

Based on the contextual realities pertaining to people in the area and their drought experiences, we argue that an accumulation of the interactive challenges from drought poses an increased risk to PLHIV in uMkhanyakude; this includes the risk of poor adherence contributing to treatment failure. Therefore, drawing on the content of our interview data and existing institutional frameworks designed for drought mitigation as well as HIV management in South Africa, we make some recommendations.

The actors mandated by the DMA to oversee drought mitigation and planning must actively prioritize and identify local drivers of vulnerability and information-sharing gaps. This would improve planning and interventions regarding droughts in the area. In uMkhanyakude, some of the local sources of vulnerability that drought can exacerbate include pervasive poverty, precarious labour, poorly maintained public water delivery systems, poor service delivery as well as poorly monitored water reservoirs. A drought management strategy that adequately addresses these issues would thus require effective collaboration with all major stakeholders, taking into planning the voices and recommendations of local stakeholders, including civil society groups.

Providing drought relief in terms of cash support or compensations, borehole installation, food banks and consistent water truck presence in communities remain commendable interventions as these will help to lessen the stress that local populations face economically and, for PLHIV, access to water to facilitate their ability to focus on treatment or to reach clinics in time.

Finally, institutional coordination for drought risk management in South Africa must transcend talk-shows or top-down processes where decisions are pre-set. Rather, collaborative efforts must be practiced more intentionally to improve both information/data sharing and inclusive and integrated actions from ground-up—a more demand-driven process—to facilitate planning and risk reduction leveraging both local knowledge and the technological strengths of institutions such as the SAWS.

## Data Availability

The data presented in this study are available on request from the corresponding author. The data are not publicly available due to privacy issues.
